# Older Adults’ Experiences in an Online Intervention for Managing Subjective Depressive Symptoms

**DOI:** 10.1093/geroni/igab046.3122

**Published:** 2021-12-17

**Authors:** Janella Hudson, Rachel Ungar, Laurie Albright, Rifky Tkatch, James Schaeffer, Ellen Wicker

**Affiliations:** 1 Optum/United Healthcare, Tampa, Florida, United States; 2 UnitedHealth Group, Minnetonka, Minnesota, United States; 3 UnitedHealth Group, Minneapolis, Minnesota, United States; 4 UnitedHealth Group, Oak Park, Michigan, United States; 5 AARP Services, Inc., Washington, District of Columbia, United States

## Abstract

Background: Many older adults struggle with late-life depression, stress, and anxiety, especially when facing age-related transitions including retirement, relocation, and the death of a spouse. Given the consequences of depression among older adults, which include higher rates of suicide, timely interventions that help to manage depressive symptoms are essential. Objective: The primary purpose of this study was to explore the perceived efficacy of an online program in improving subjective depressive feelings. Methods: Older adult participants were recruited for semi-structured interviews (n = 24) in a web-based intervention that included interactive games and activities undergirded by a cognitive behavioral therapy (CBT) approach. Participants were asked to provide feedback about program features, including weekly module content, games, interactive activities and community interactions, and any perceived effects on their health behaviors and/or emotional well-being. Participants’ responses were analyzed using qualitative content analysis. Results: Participants reported several gains, including developing the habit of forming ongoing, incremental goals, achieving wellness-related goals, and experiencing an overall positive shift in perspective. In addition, participants reported feeling greater gratitude, increased positivity, and improvement in mood. Featured games and activities helped to promote stress relief and entertainment, and mindfulness exercises were cited as the most helpful and/or enjoyable among participants. Participants expressed a preference for program content related to aging and aging-related transitions. Conclusions: This study demonstrated feasibility of an interactive web-based intervention for older adults with subjective depressive feelings, while also providing important findings about users’ preferences for personalized, aging-related feedback.

